# Mapping Comorbidities in Patients With Low Back Pain—A Systematic Review

**DOI:** 10.1002/pri.70109

**Published:** 2025-09-19

**Authors:** Jacob S. Gandløse, Caroline W. M. Sørensen, Cecilie T. Hemmingsen, Kasper W. Larsen, Thorvaldur S. Palsson

**Affiliations:** ^1^ Department of Physiotherapy and Occupational Therapy Aalborg University Hospital Aalborg Denmark; ^2^ Department of Clinical Medicine Aalborg University Aalborg Denmark; ^3^ Department of Rehabilitation and Health Promotion Research Faculty of Health Science VIA University College Aarhus Denmark

**Keywords:** comorbidities, low back pain, observational studies, prevalence, systematic review

## Abstract

**Background and Purpose:**

Low back pain (LBP) frequently co‐occurs with other health conditions, but there is no clear consensus on which comorbidities are most prevalent. This limits understanding of the broader burden and challenges clinical practice.

**Methods:**

Systematic searches were conducted in seven databases, including PubMed, Embase, CINAHL, PsycINFO, PEDro, Rehabilitation and Sports Medicine Source, and Cochrane Library. Observational studies on adults (≥ 16 years) with nonspecific LBP and at least three comorbidities were included. Data were extracted, tabulated, and quality assessed using CASP checklists and GRADE classifications.

**Results:**

Nine peer‐reviewed studies were included. The prevalence of at least one comorbidity ranged from 49% to 92%, with hypertension, osteoarthritis, and chronic pain elsewhere in the body being the most proportionally prevalent comorbidities in individual study populations. Across all studies, hypertension, diabetes, osteoarthritis, asthma, and depression were the most frequently reported comorbidities. Comorbidity assessment, population characteristics, and prevalence varied significantly. Certainty in the prevalence of comorbidities varied from “very low” to “moderate.”

**Discussion:**

Comorbidities are highly prevalent in adults with LBP. The included studies varied considerably in comorbidity assessment, reporting, and study populations, where sociodemographic factors like sex, age, marital status, education, and employment likely influenced comorbidity prevalence and types. Findings emphasize the need for standardized assessment methods and tailored physiotherapy approaches to address the diverse needs of this population.

## 1 Background

Low back pain (LBP) is a leading cause of disability and imposes a significant economic burden through healthcare costs, productivity loss, and sickness absence (Ferrari et al. 2024). Part of this substantial individual and societal burden may arise from the frequent coexistence of other health conditions alongside LBP (Hartvigsen et al. 2013), a phenomenon often referred to as multimorbidity (Skou et al. 2022). Multimorbidity differs from comorbidity in how a patient's health conditions are prioritized and managed. In comorbidity, one index disease is the primary focus, with other conditions treated based on their impact on the index condition. In contrast, multimorbidity involves no index condition; all health conditions are considered equally important, and treatment is based on the patient's overall needs and the treatment goals most important to them (Skou et al. 2022).

Individuals experiencing musculoskeletal pain and concurrent health conditions tend to report higher pain levels, greater limitations in daily activities, and poorer treatment outcomes compared to those without multimorbidity (Legha et al. 2020; Pihl et al. 2021; van Dijk et al. 2008). Furthermore, multimorbidity in general can negatively influence quality of life, increase healthcare utilization, and contribute to polypharmacy, which in turn heightens the complexity of clinical decision‐making (Skou et al. 2022).

Adding to this complexity is the underrepresentation of patients with multimorbidity in research. This often occurs through systematic exclusion of patients with comorbidities in studies or through selection bias driven by factors such as high treatment burden and low health literacy (Gandløse et al. 2025; He et al. 2020). Clearly, this lack of representation limits the generalizability of research findings to the broader patient population (Gluud 2006). In fact, this is reflected in a scoping review highlighting that clinical practice lacks guidance on managing comorbidities in chronic spinal pain, as guidelines often overlook these complexities (Gandløse et al. 2024). Ultimately, this may result in patients with multimorbidity often feeling that their overall health is inadequately addressed, with treatments primarily focusing on individual conditions rather than the cumulative challenges they face (Bair et al. 2009; Makris et al. 2015). Consequently, treatment is frequently perceived as ineffective and incoherent (van der Aa et al. 2017).

To improve the spinal pain management in multimorbidity, the NICE guidelines recommend a holistic, patient‐centered approach that addresses the full range of health conditions and their interactions (Farmer et al. 2016). However, excluding patients with comorbidities from studies creates significant gaps in understanding the extent and profile of comorbidities in individuals with treatment‐requiring LBP. The burden and implications of comorbidities in this group therefore remain poorly characterized. Previous research highlights that studies on multimorbidity employ varied data collection methods and focus on different conditions, complicating the identification of prevalent comorbidities in specific populations (Ho et al. 2021). The aim of this systematic review was to provide a broad overview of the existing literature by mapping the types and prevalence of comorbidities, the proportion of patients with LBP who had at least one comorbidity, the data collection methods, and the demographic characteristics of study populations. By synthesizing this evidence, the review seeks to improve understanding of the overall burden of low back pain, inform clinical management, and identify priorities for future research.

## 2 Methods

### 2.1 Study Design and Search Strategy

This systematic review followed the Preferred Reporting Items for Systematic Reviews and Meta‐Analyses (PRISMA) (see Supporting Information S1: Appendix 1). An initial, free‐text search was conducted in PubMed and Google Scholar using terms such as “*low back pain*,” “*comorbidities*,” and “*multimorbidity*.” The search strategy was refined by incorporating thesaurus headings (e.g., MeSH and CINAHL) and free‐text terms related to LBP and comorbidities. Citation tracking from reference lists was also used to gather additional relevant studies. The final systematic search, conducted on November 1, 2024, included the following databases: PubMed, Embase, CINAHL Complete, APA PsycINFO, PEDro, and Rehabilitation and Sports Medicine Source. The Cochrane Library was searched to identify relevant systematic reviews. The search strategy focused on two main elements: the population (people with LBP) and comorbidities. The search process was conducted in collaboration with an experienced research librarian, and a pilot search was performed to refine the search terms. For a detailed overview of the search strategy in each database, see Supporting Information S2: Appendix 2. Following prior recommendations (Peters et al. 2022), a flow diagram was developed to outline the process of screening, assessing eligibility, and including studies in the review.

### 2.2 Eligibility Criteria

For this systematic review, eligibility criteria were established to identify relevant studies. Included studies had to focus on nonspecific LBP as the primary diagnosis. Since previous work has shown that multimorbidity can also affect younger populations (Barnett et al. 2012), no upper or lower age limits were applied to study eligibility. The duration of pain was not a limiting factor and could include acute, subacute, or chronic pain. Eligible studies had to explicitly aim to determine the prevalence of at least three concurrent comorbidities, such as, but not limited to, depression, anxiety, musculoskeletal pain in other body regions, chronic obstructive pulmonary disease, or type 2 diabetes mellitus. Only observational studies, including longitudinal, cross‐sectional, and cohort designs from all clinical settings (primary, secondary, and tertiary care), were considered. Studies were included if published in English, Danish, Norwegian, or Swedish, with full‐text availability in these languages. To provide an overview, studies that were assessed for eligibility but did not meet the inclusion criteria are presented separately in Supporting Information S3: Appendix 3.

### 2.3 Selection Process

All studies identified through the literature search were uploaded to a web‐based platform for systematic reviews and meta‐analyses (www.rayyan.ai), following previously used methods (Ouzzani et al. 2016). After removing duplicates, relevant studies were selected through a two‐stage screening process conducted by all three reviewers (C.W.M.S., C.T.H., and K.W.L.), who were blinded to each other's decisions. The screening process began with a pilot screening, where all three reviewers assessed the titles and abstracts of the first 100 studies to identify whether potential articles met the inclusion and exclusion criteria. The results from all reviewers were compared to ensure consensus.

The screening process was carried out in two stages: an initial review of titles and abstracts, followed by a full‐text evaluation. The first stage involved screening studies based on titles and abstracts. Studies were excluded from further screening if (1) the abstract was not available, (2) the study was in a language other than Danish, Norwegian, Swedish, or English, or (3) the title and abstract clearly indicated that the study did not meet the inclusion criteria. In the second stage of screening, full‐text articles were assessed for eligibility and excluded in case of the following: (1) The patient population did not match the focus of the review (e.g., patients without LBP or without at least two additional chronic conditions), (2) The study design was unsuitable (e.g., not observational studies investigating the prevalence of comorbidities in the target population), or (3) The outcomes were inappropriate (e.g., comorbidities were not clinically diagnosed but instead defined by symptoms or cutoff scores on questionnaires). If consensus could not be reached between reviewers, a final decision was made by a fourth reviewer (J.S.G.). To ensure no relevant studies were missed, the reference lists of the included studies were screened for studies meeting the eligibility criteria.

### 2.4 Quality Assessment

Three reviewers (C.W.M.S., C.T.H., and K.W.L.) independently assessed the methodological quality of the included studies using the Critical Appraisal Skill Programme (CASP) checklists for cohort and cross‐sectional studies (Critical Appraisal Skills Programme, 2023b; Critical Appraisal Skills Programme 2023a). Disagreements were resolved through discussion or by involving a fourth reviewer (J.S.G.). Subsequently, studies were assigned an overall assessment inspired by the approach used in the Grading of Recommendations, Assessment, Development, and Evaluation (GRADE) system (Balshem et al. 2011), based on evaluations using the CASP checklist. Studies were categorized into four groups reflecting their certainty regarding the accuracy of prevalence estimates: “*Very low certainty*,” “*Low certainty*,” “*Moderate certainty*,” and “*High certainty*.” Certainty ratings were based on author consensus and guided by the GRADE approach, taking into account study quality (assessed via CASP), consistency, and directness. Justifications for each rating are detailed in Supporting Information S5: Appendix 5.

### 2.5 Synthesis of Results

Relevant data were collected, organized, and systematically summarized in tables using Microsoft Excel 2016 (*Microsoft, Washington, USA*). The data extraction included study design, population characteristics (age, sex), country of origin, data sources (e.g., patient‐reported, registry, or database), sample size, and the five most prevalent comorbidities for each study. Additionally, all reported comorbidities and further demographic information, such as educational level, employment status, BMI, smoking habits, duration of LBP, and marital status, were extracted and compiled in Supporting Information S4: Appendix 4. Based on these data, the five most frequently reported comorbidities across studies across all studies were identified. Finally, the proportion of patients with LBP who had at least one comorbidity was also extracted to provide an overview of patients with multimorbidity.

## 3 Results

**FIGURE 1:**
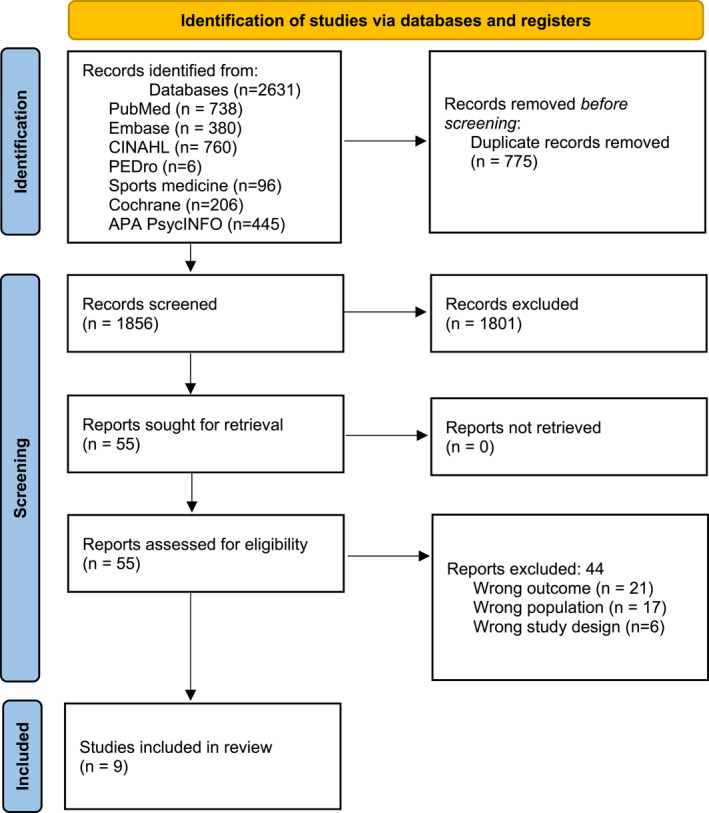
PRISMA flowchart demonstrating the findings in each step of the screening process. A PRISMA flowchart depicting the study selection process in the systematic review. The diagram details the number of records identified through database searches, the number screened after duplicate removal, and the reasons for exclusion at different stages. It further shows the number of full‐text articles assessed for eligibility and the final number of studies included in the review.

A systematic literature search across all databases identified 2631 studies. Of these, 775 were duplicates. The remaining 1856 studies were screened based on title and abstract. A total of 1801 studies were excluded, primarily due to incorrect study design and/or population. Fifty‐five studies proceeded to full‐text screening, where 44 studies were excluded. This led to a final inclusion of nine studies (Figure 1). See Supporting Information S3: Appendix 3 for a list of studies excluded during full‐text screening.

### 3.1 Characteristics of Included Studies

The included studies consisted of two cohort studies (Leopoldino et al. 2020; Rafn et al. 2023) and seven cross‐sectional studies (Bartholomeeusen et al. 2012; Gore et al. 2012; Marunica Karšaj et al. 2023; Ramanathan et al. 2018; Ritzwoller et al. 2006; Schneider et al. 2007; Von Korff et al. 2005). The studies represented populations from Denmark, USA, Germany, Australia, Brazil, Belgium, and Croatia (Table 1). Six studies examined the association between LBP and comorbidities, finding that individuals with LBP had more comorbidities compared with a control sample (Bartholomeeusen et al. 2012; Gore et al. 2012; Marunica Karšaj et al. 2023; Ritzwoller et al. 2006; Schneider et al. 2007).

**TABLE 1:** Overview of included studies.

Study design	Country and data source	Patient characteristics	Most common comorbidities (%)
Rafn et al. (2023) Prospective cohort study	Country: Denmark Data source: Patient‐reported data via a questionnaire asking participants whether a medical doctor had ever told them they have or have had specific chronic conditions	*n*: 2083	Hypertension (19%)
		Age: ≥ 18 years	Osteoarthritis (14.6%)
		Mean (SD): 46.07 (13.50)	Migraine (8.7%)
		Men: 55.9%	Asthma (8.6%)
		Women: 44.1%	Psoriasis (5%)
Leopoldino et al. (2020) Prospective cohort study	Country: Brazil Data source: Patient‐reported via “Self‐Administered Comorbidities Questionnaire”	*n*: 602	Hypertension (70.9%)
		Age: ≥ 55 years	Osteoarthritis (46.9%)
		Mean (SD): 67.7 (7.0)	Gastrointestinal disease/ulcer (33.6%)
		Men: 15.1%	Depression (32.7%)
		Women: 84.9%	Diabetes (23.8%)
Von Korff et al. (2005) Cross sectional study	Country: USA Data source: “National Comorbidity Survey Replication”—A face‐to‐face household survey	*n*: 1668	Osteoarthritis (51%)
		Age: ≥ 18 years	Hypertension (26.6%)
		Range: 18–60+	Other chronic pain (18.9%)
		Men: 39.8%	Asthma (16.9%)
		Women: 60.2%	Other headaches (14.6%)
Marunica Karšaj et al. (2023) Cross sectional study	Country: Croatia Data source: Population‐based cross‐sectional “European Health Interview Survey”	*n*: 268	Chronic neck pain (65.7%)
		Age: ≥ 18 years	Hypertension (42.9%)
		Median: 60 years	Allergies (23.9%)
		Men: 41%	Osteoarthritis (19.8%)
		Women: 59%	Urinary incontinence (19.0%)
Ramanathan et al. (2018) Cross sectional study	Country: Australia Data source: “CareTrack Australia” (consisting of medical record data and patient‐reported data)	*n*: 164	Hypertension (28.7%)
		Age: ≥ 18 years	Osteoarthritis (25.6%)
		Range: 18–75+	Hyperlipidemia (15.9%)
		Men: 46.5%	Dyspepsia (13.4%)
		Women: 53.5%	Depression (9.8%)
Schneider et al. (2007) Cross sectional study	Country: Germany Data source: “National Health Survey in Germany” consisting of self‐reported and medical examinations	*n*: 2475	Osteoarthritis (33%)
		Age: ≥ 18 years	Gastritis (30%)
		Range: 19–79	Hypertension (26%)
		Men: 42.8%	Varicose veins (25%)
		Women: 57.2%	Migraine (23%)
Gore et al. (2012) Cross sectional study	Country: USA Data source: The “LifeLink Health Plan Claims Database”	*n*: 101,294	Chronic back and neck pain (besides LBP) (43%)
		Age: ≥ 18 years	Other musculoskeletal pain conditions (41%)
		Mean (SD): 47.2 (11.6)	Rheumatism (besides LBP) (40%)
		Men: 45%	Arthritis and joint diseases (34%)
		Woman: 55%	Neuropathic back and neck pain (besides LBP) (34%)
Ritzwoller et al. (2006) Cross sectional study	Country: USA Data source: Registry data from “Kaiser Permanente Colorado”	*n*: 16,567	Hypertension (19.4%)
		Age: ≥ 18 years	Inflammation (15.6%)
		Range: 18–85+	Heart disease (14%)
		Mean (SD): 51.09 (n/a)	Anxiety (12.2%)
		Men: 46.3%	Gastrointestinal disease (12.1%)
		Women: 53.7%	
Bartholomeeusen et al. (2012) Cross sectional study	Country: Belgium Data source: “Intego Database”	*n*: 3851	Neck pain (5.7%)
		Age: ≥ 18 years	Shoulder syndrome (0.7%)
		Range: 25–74	Other musculoskeletal disorders (0.4%)
		Men: 49.4%	Hip osteoarthritis (0.2%)
		Women: 50.6%	Depression (0.2%)

Abbreviation: *n*, number of participants.

### 3.2 Data Collection and Reporting

The proportion of patients with LBP who had at least one comorbidity ranged from 49% to 92% (Rafn et al. 2023; Schneider et al. 2007). Three studies reported method‐dependent estimates, ranging from 68.7%–87.1%, 61.1%–68.7%, and 62%–81%, respectively (Marunica Karšaj et al. 2023; Ramanathan et al. 2018; Von Korff et al. 2005). One study only reported the proportion of patients with at least three comorbidities, with 41.9% meeting this criterion (Leopoldino et al. 2020). Three studies did not report this proportion (Bartholomeeusen et al. 2012; Gore et al. 2012; Ritzwoller et al. 2006).

The collection of comorbidities varied across studies in terms of the specific comorbidities identified, the total number of comorbidities, and the methods used for data collection. The number of comorbidities ranged from 5 to 31. For instance, Bartholomeeusen et al. (2012) reported 5 comorbidities (“neck pain,” “shoulder syndrome,” “musculoskeletal diseases,” “hip osteoarthritis,” “depression”), while Schneider et al. (2007) reported the highest number, with 31 comorbidities, which included conditions such as osteoarthritis, cardiovascular diseases, diabetes, respiratory diseases, mental health disorders, and various musculoskeletal and neurological conditions.

Three studies focused solely on physical comorbidities (Rafn et al. 2023; Schneider et al. 2007; Von Korff et al. 2005), while six examined both physical and psychological comorbidities (Bartholomeeusen et al. 2012; Gore et al. 2012; Leopoldino et al. 2020; Marunica Karšaj et al. 2023; Ramanathan et al. 2018; Ritzwoller et al. 2006). No studies reported only psychological comorbidities. The included studies focused primarily on musculoskeletal pain conditions, with a significant proportion of the reported comorbidities being related to pain (Supporting Information S4: Appendix 4). Two studies gathered information on comorbidities using patient‐reported questionnaires or interviews (Marunica Karšaj et al. 2023; Rafn et al. 2023). Five studies gathered information on comorbidities using databases or registries (Bartholomeeusen et al. 2012; Gore et al. 2012; Leopoldino et al. 2020; Ritzwoller et al. 2006; Von Korff et al. 2005). Two studies used both patient‐reported questionnaires and databases to collect data (Ramanathan et al. 2018; Schneider et al. 2007).

### 3.3 Most Prevalent Comorbidities Across Studies

The five most proportionally prevalent comorbidities within each study are presented in Table 1. Across all included studies, hypertension (7 studies), diabetes (7 studies), osteoarthritis (7 studies), asthma (6 studies), and depression (6 studies) were the most frequently documented comorbidities (Supporting Information S4: Appendix 4).

### 3.4 Sociodemographic Trends in Included Studies

Nine studies focused on populations aged 18 years and older (Bartholomeeusen et al. 2012; Gore et al. 2012; Marunica Karšaj et al. 2023; Rafn et al. 2023; Ramanathan et al. 2018; Ritzwoller et al. 2006; Schneider et al. 2007; Von Korff et al. 2005), while one study examined a population aged 55 years and older (Leopoldino et al. 2020). The maximum specified age limit was +85 years (Ritzwoller et al. 2006). Eight studies reported a higher prevalence of LBP in the female population (Bartholomeeusen et al. 2012; Gore et al. 2012; Leopoldino et al. 2020; Marunica Karšaj et al. 2023; Ramanathan et al. 2018; Ritzwoller et al. 2006; Schneider et al. 2007; Von Korff et al. 2005), while one study indicated a higher prevalence in men with LBP (Rafn et al. 2023). Information on sociodemographic factors, including sex, age, education, employment, and marital status, varied across the reviewed studies (Table 1 and Supporting Information S4: Appendix 4). Regarding sex distribution, several studies showed a relatively balanced representation (Table 1). However, Leopoldino et al. (2020) had a greater representation of female participants (female: 84.9%, male: 15.1%). The age distribution varied considerably across studies. Rafn et al. (2023) and Gore et al. (2012), included a broad age range, with participants aged 18 years and older, and mean ages around 46–47 years. In contrast, other studies, such as Leopoldino et al. (2020), focused specifically on older populations, with a mean age of 67.7 years. Additionally, the age ranges varied, from studies like Schneider et al. (2007), which included participants aged 19–79 years, to those like Ramanathan et al. (2018), which included participants aged 18–75 years.

Education level was assessed in seven studies, with participants generally categorized into low, middle, or higher education. For example, Rafn et al. (2023) found that 42% of participants had a low level of education, while 11.5% had a university education.

Employment was reported in most studies, with notable variation. For instance, Schneider et al. (2007) reported that only 1/3 of participants were employed, whereas Rafn et al. (2023) reported that 82.1% of participants were employed. Finally, marital status was reported in three studies, with little variation observed (Supporting Information S4: Appendix 4).

### 3.5 Quality Assessment

The results of the quality assessment via CASP are presented in Tables 2 and 3. The most common reason for scoring a study as “*no*” or “*can't tell*” on individual questions, thus assessing it as having a risk of bias, was an insufficient or unclear description of items 3, 4, 6, and 10. These items include participant recruitment and exposure, reporting of information/outcomes, participation rate, and generalizability to the broader population. According to the assessment of certainty in prevalence estimates, three of the included studies were classified as having “*very low certainty*” (Leopoldino et al. 2020; Ramanathan et al. 2018; Von Korff et al. 2005), while four as having “*low certainty*” (Gore et al. 2012; Rafn et al. 2023; Ritzwoller et al. 2006; Schneider et al. 2007), and two as having “*moderate certainty*” (Bartholomeeusen et al. 2012; Marunica Karšaj et al. 2023) (Supporting Information S5: Appendix 5).

**TABLE 2:** CASP checklist for cohort studies.

	1	2	3	4	5a	5b	6a	6b	7	8	9	10	11	12
Rafn et al. (2023)	+	+	+	%	+	+	+	+	+	+	+	?	+	+
Leopoldino et al. (2020)	+	+	%	+	+	+	+	+	+	+	+	+	+	+

*Note:* Responses were categorized as “+” (Yes), “%” (No), and “?” (Can't tell). “+” indicates that the criterion was clearly met, “%” indicates that it was not met, and “?” reflects insufficient information to make a judgment.

**TABLE 3:** CASP checklist for cohort studies.

	1	2	3	4	5	6	7	8	9	10	11
Von Korff et al. (2005)	+	+	+	%	+	+	+	+	+	%	?
Marunica Karšaj et al. (2023)	+	+	+	%	+	+	+	+	+	+	+
Ramanathan et al. (2018)	+	+	+	+	+	%	+	%	+	+	+
Schneider et al. (2007)	+	+	+	%	+	+	+	+	+	+	+
Gore et al. (2012)	+	+	%	+	+	+	+	+	+	−	+
Ritzwoller et al. (2006)	+	+	+	+	+	+	+	+	+	−	+
Bartholomeeusen et al. (2012)	+	+	+	+	+	+	+	+	+	?	?

*Note:* Responses were categorized as “+” (Yes), “%” (No), and “?” (Can't tell). “+” indicates that the criterion was clearly met, “%” indicates that it was not met, and “?” reflects insufficient information to make a judgment.

## 4 Discussion

This systematic review provided a broad overview of the existing literature by mapping the types and prevalence of comorbidities, the data collection methods, and the demographic characteristics of study populations. Nine peer‐reviewed studies were included. The prevalence of at least one comorbidity ranged from 49% to 92%, with hypertension (19%–70.9%), osteoarthritis (14.6%–46.9%), and chronic pain elsewhere in the body (5.4%–65.7%) being the most proportionally prevalent comorbidities within the individual study populations (Table 1). When considering comorbidities reported across all included studies, hypertension (7 studies), diabetes (7 studies), osteoarthritis (7 studies), asthma (6 studies), and depression (6 studies) were the most frequently documented. All included studies were evaluated using CASP checklists for cohort or cross‐sectional studies. Three of the included studies were classified as having “*very low certainty*” (Leopoldino et al. 2020; Ramanathan et al. 2018; Von Korff et al. 2005), while four as having “*low certainty*” (Gore et al. 2012; Rafn et al. 2023; Ritzwoller et al. 2006; Schneider et al. 2007), and two as having “*moderate certainty*” (Bartholomeeusen et al. 2012; Marunica Karšaj et al. 2023) regarding the certainty of prevalence estimates (Supporting Information S5: Appendix 5).

### 4.1 Great Diversity in Methodology and Comorbidity Prevalence Across Studies

Across the included studies, population characteristics (e.g., age), comorbidity collection, and reported prevalence varied significantly. It is well‐documented that the prevalence of comorbidities varies depending on whether self‐reported or administrative data is used (Jensen et al. 2023; Selçuk et al. 2021). This discrepancy was evident in Ramanathan et al. (2018) where 81% met the criteria for multimorbidity based on patient‐reported data, compared to only 62% when using medical records. Such methodological differences likely contribute to the wide variation in reported prevalence estimates. For instance, depression ranged from 0.2% in a cohort with mean age 45 (Bartholomeeusen et al. 2012) to 32.7% in an older cohort (mean age 60) (Schneider et al. 2007). Similarly, the occurrence of anxiety was reported to lie between 8% and 19% (Bartholomeeusen et al. 2012; Gore et al. 2012), with higher rates found in the older population. Similarly, osteoarthritis prevalence ranged from 5% to 22.4% (Gore et al. 2012; Schneider et al. 2007), with higher occurrence in older populations. Collectively, these differences demonstrated the influence of age on comorbidity prevalence, with older individuals showing significantly higher rates of depression, anxiety, osteoarthritis, and other comorbidities (Skou et al. 2022). Clearly, this underscores the clear need for age stratification and consensus on the specific comorbidities considered across studies. Many studies use broad and unspecific terms, complicating comparisons. For instance, terms such as “*musculoskeletal diseases*” (Bartholomeeusen et al. 2012) and “*neuropathic conditions*” (Gore et al. 2012) lack clear definitions, leading to inconsistencies in how these conditions are identified and reported.

Several studies were excluded from this review because of their focus on health complaints or symptoms rather than formal diagnoses of comorbidities (Supporting Information S3: Appendix 3). The lack of standardized definitions and criteria further complicates comparisons. Recent efforts such as the Delphi consensus on multimorbidity aim to standardize comorbidity classification, ensuring more accurate and comparable prevalence data across populations (Ho et al. 2022), and could also be applied in this context to ensure comparability.

### 4.2 Managing Comorbidities in LBP: The Need for Adapted Treatment Strategies in Modern Healthcare

The included studies demonstrate that individuals with LBP often have co‐occurring chronic conditions, but given the observational nature of the data, it is not possible to determine causality (Bartholomeeusen et al. 2012; Gore et al. 2012; Marunica Karšaj et al. 2023; Ritzwoller et al. 2006; Schneider et al. 2007). However, various explanatory mechanisms have been proposed in the literature to help understand why certain comorbidities are more prevalent among individuals with LBP. Some may be consequences of the pain itself or its treatment—such as gastrointestinal disorders resulting from long‐term use of non‐steroidal anti‐inflammatory drugs (Schneider et al. 2007), while others, including cardiovascular and respiratory diseases, may be driven by shared lifestyle‐related risk factors such as physical inactivity, smoking, and obesity (Ramanathan et al. 2018; Schneider et al. 2007). In addition, chronic conditions such as depression, osteoarthritis, asthma, and diabetes have been independently associated with LBP (Ferreira et al. 2013). Sex‐ and education‐related differences, as observed in several included studies, may also reflect broader biological, psychological, and sociocultural influences (Schneider et al. 2007) as well as disparities in health literacy and healthcare access (Leopoldino et al. 2020).

Although the precise nature of these associations remains speculative, recognizing them is essential when designing interventions. Recent evidence indicates that managing comorbidities alongside LBP can improve patient outcomes (Gandløse et al. 2024). Additionally, the value of exercise and physical activity has been well‐documented for a wide range of health conditions and are generally considered beneficial and safe for individuals with multimorbidity (Bricca et al. 2020; Pedersen and Saltin 2015). In line with this, NICE guidelines on multimorbidity recommend a coordinated, patient‐centered approach focused on maintaining function and quality of life across all conditions (Farmer et al. 2016). The physiotherapy profession, with its expertise in musculoskeletal treatment and the integration of physical activity, may reduce the overall treatment burden by tailoring exercise programs to address both pain and comorbid conditions (Skou et al. 2025). This holistic approach may enable physiotherapists to contribute significantly to the coordination of care, promoting better health outcomes for patients with multiple conditions (Skou et al. 2022). However, healthcare professionals frequently feel unqualified to manage musculoskeletal pain in individuals with comorbidities (Rømer et al. 2025), highlighting the need for greater knowledge and expertise in this area. Research on how to manage LBP and comorbidities is therefore essential to better reflect the patient population and support physiotherapists in delivering holistic care.

### 4.3 Methodological Considerations and Limitations

This systematic review provides valuable insights into the prevalence of comorbidities among individuals with LBP and their potential impact on treatment pathways. However, several methodological considerations and limitations should be acknowledged.

One strength of this review is the comprehensive approach to identifying and synthesizing relevant studies. The inclusion of a wide range of studies allowed for a thorough exploration of the relationship between multimorbidity and LBP. A key advantage of this approach is the inclusion of observational studies, which provide a broader representation of the general population, particularly when it comes to LBP and comorbidities. By focusing on observational designs, such as cross‐sectional and cohort studies, the review managed to capture trends and associations within large populations that are often difficult to identify in more selective experimental studies, such as randomized controlled trials. These designs are particularly suited for examining the prevalence and relationships between LBP and comorbidities in diverse populations, offering insights into real‐world scenarios where controlled conditions are not always feasible. However, a key limitation of this review is that not all studies clearly indicated whether the comorbidities described were diagnosed as diseases or simply health complaints. This lack of clarity can impact both the internal and external validity of the findings, as the definition of comorbidities can vary across studies. Another important limitation pertains to the assessment of certainty in the comorbidity estimates. Given that no comprehensive tools currently exist to assess the quality of prevalence studies (Kelly et al. 2024), the approach in this study was inspired by GRADE's rating system. While this method provides a useful framework for evaluating certainty, the lack of a dedicated tool for prevalence studies limits the robustness of the certainty assessment. Most included studies were conducted in high‐income countries, which may limit the generalizability of findings to other settings. Additionally, the socio‐demographic differences among the included studies, which came from different countries with varying healthcare systems and population compositions, may affect the generalizability of the results. These differences suggest that the findings may not be fully extrapolated to all populations. Interpretation should therefore be approached with caution, particularly when applying the results to different healthcare settings or demographic groups. Finally, this study was not pre‐registered prior to designing the search strategy and summarizing the findings. Nevertheless, the authors followed strict inclusion and exclusion criteria as well as using recognized assessment tools to evaluate the quality of the included literature. This does however not negate the importance of pre‐registering reviews of the existing literature, which is clearly a limitation of this review.

## 5 Implications for Physiotherapy Practice

This review highlights the high prevalence of comorbidities in adults with LBP, underscoring the need for a holistic and individualized physiotherapy approach. Physiotherapists play a key role in supporting physical activity and reducing treatment burden, though designing effective interventions can be challenging. The variability in how comorbidities are assessed across studies emphasizes the need for thorough evaluations and flexible treatment strategies. Tailoring exercise programs to address both LBP and comorbidities may improve care coordination and enhance outcomes. However, further research is essential to better understand these relationships. Additionally, increasing the focus on multimorbidity in physiotherapy education is crucial to ensure clinicians are equipped to deliver comprehensive, patient‐centered care.

## Author Contributions

Concept/idea/research design: Jacob S. Gandløse and Thorvaldur S. Palsson. Acquisition of data: Caroline W. M. Sørensen, Kasper W. Larsen, and Cecilie T. Hemmingsen. Analysis and interpretation of data: Caroline W. M. Sørensen, Kasper W. Larsen, and Cecilie T. Hemmingsen. Writing/review/editing of manuscript: Caroline W. M. Sørensen, Kasper W. Larsen, Cecilie T. Hemmingsen, Jacob S. Gandløse and Thorvaldur S. Palsson. Final approval of the manuscript: Jacob S. Gandløse and Thorvaldur S. Palsson.

## Ethics Statement

The authors have nothing to report.

## Consent

The authors have nothing to report.

## Conflicts of Interest

The authors declare no conflicts of interest.

## Permission to Reproduce Material From Other Sources

The authors have nothing to report.

## Supporting information


**Supporting Information S1**: PRISMA 2020 checklist.

**Supporting Information S2**: Search strategy for all the included databases.

**Supporting Information S3**: List of excluded studies reviewed for eligibility.

**Supporting Information S4**: Extended information of included studies.

**Supporting Information S5**: Level of certainty in comorbidity prevalence estimates in included studies.

## Data Availability

All data included in this systematic review are publicly available from the original studies cited in the manuscript. No new data were generated for this review.
