# Supplementary material for: Mapping Comorbidities in Patients With Low Back Pain—A Systematic Review
Source: Physiother Res Int. 2025 Sep 19;30(4):e70109. doi: 10.1002/pri.70109 (PMC12449286; doi:10.1002/pri.70109)
Supplement: Supplementary file 2 — Supporting Information S2: Search strategy for all the included databases. [file PRI-30-e70109-s005.docx]

**Appendix 2:** Search strategy for all the included databases

| **Database** | **Block 1 (Low Back Pain)** | **Block 2 (Comorbidity)** | **Number of hits** |
| --- | --- | --- | --- |
| **PubMed** | Low Back Pain (MeSH) "Low back pain" (free text) "Lumbar pain" (free text) | Comorbidity (MeSH) Comorbidity (free text) "Co-morbid" (free text) Comorbid (free text) | 738 |
| **Embase** | Low Back Pain (EmTree) 'Low Back Pain' (free text, title, and abstract) Lumbar pain (free text, title, and abstract) | Comorbidity (EmTree) Comorbidity (free text, title, and abstract) 'Co-morbid' (free text, title, and abstract) Comorbid (free text, title, and abstract) | 380 |
| **CINAHL** | Low Back Pain (subject heading) "Low Back Pain" (free text) "Lumbar pain" (free text) | Comorbidity (subject heading) Comorbidity (free text) "Co-morbid" (free text) Comorbid (free text) | 760 |
| **Rehabilitation and Sport Medicine** | Low Back Pain (Thesaurus) "Low Back Pain" (free text) "Lumbar pain" (free text) | Comorbidity (Thesaurus) Comorbidity (free text) "Co-morbid" (free text) Comorbid (free text) | 96 |
| **PEDro** | Lumbar spine, sacro-iliac joint or pelvis | Comorbidity | 6 |
| **Cochrane Library** | Low Back Pain (MeSH) "Low Back Pain" (free text) "Lumbar pain" (free text) | Comorbidity (MeSH) Comorbidity (free text) "Co-morbid" (free text) Comorbid (free text) | 206 |
| **APA PsycInfo** | Back pain (Thesaurus) "Back pain" (free text) "Low Back Pain" (free text) "Lumbar pain" (free text) | Comorbidity (Thesaurus) Comorbidity (free text) "Co-morbid" (free text) Comorbid (free text) | 445 |
