# Supplementary material for: Mapping Comorbidities in Patients With Low Back Pain—A Systematic Review
Source: Physiother Res Int. 2025 Sep 19;30(4):e70109. doi: 10.1002/pri.70109 (PMC12449286; doi:10.1002/pri.70109)
Supplement: Supplementary file 3 — Supporting Information S3: List of excluded studies reviewed for eligibility. [file PRI-30-e70109-s002.docx]

**Appendix** **3:** List of excluded studies reviewed for eligibility

### Wrong outcome (comorbidities defined based on questionnaire cut-off values): [1–21]

### Wrong population: [22–38]

### Wrong study design: [39–44]

**References**

1. Park HJ, Choi JY, Lee WM, Park SM. Prevalence of chronic low back pain and its associated factors in the general population of South Korea: a cross-sectional study using the National Health and Nutrition Examination Surveys. J Orthop Surg Res. 2023 Jan 11;18(1):29.

2. Licciardone JC, Pandya V. Prevalence and Impact of Comorbid Widespread Pain in Adults with Chronic Low Back Pain: A Registry-Based Study. J Am Board Fam Med. 2020 Jul;33(4):541–8.

3. Ramond-Roquin A, Pecquenard F, Schers H, Van Weel C, Oskam S, Van Boven K. Psychosocial, musculoskeletal and somatoform comorbidity in patients with chronic low back pain: original results from the Dutch Transition Project. Family Practice. 2015 Jun 1;32(3):297–304.

4. Nordstoga AL, Nilsen TIL, Vasseljen O, Unsgaard-Tøndel M, Mork PJ. The influence of multisite pain and psychological comorbidity on prognosis of chronic low back pain: longitudinal data from the Norwegian HUNT Study. BMJ Open. 2017 May;7(5):e015312.

5. Hung CI, Liu CY, Fu TS. Depression: An important factor associated with disability among patients with chronic low back pain. Int J Psychiatry Med. 2015 Apr;49(3):187–98.

6. Reme SE, Tangen T, Moe T, Eriksen HR. Prevalence of psychiatric disorders in sick listed chronic low back pain patients. European Journal of Pain. 2011 Nov;15(10):1075–80.

7. Mirzamani SM, Sadidi A, Sahrai J, Besharat MA. Anxiety and Depression in Patients with Lower Back Pain. Psychol Rep. 2005 Jun;96(3):553–8.

8. Kääriä S, Solovieva S, Leino‐Arjas P. Associations of low back pain with neck pain: A study of industrial employees with 5‐, 10‐, and 28‐year follow‐ups. European Journal of Pain. 2009 Apr;13(4):406–11.

9. Park SM, Kim HJ, Jang S, Kim H, Chang BS, Lee CK, et al. Depression is Closely Associated With Chronic Low Back Pain in Patients Over 50 Years of Age: A Cross-sectional Study Using the Sixth Korea National Health and Nutrition Examination Survey (KNHANES VI-2). Spine. 2018 Sep 15;43(18):1281–8.

10. IJzelenberg W, Burdorf A. Impact of musculoskeletal co-morbidity of neck and upper extremities on healthcare utilisation and sickness absence for low back pain. Occup Environ Med. 2004 Oct;61(10):806–10.

11. Fujii T, Oka H, Katsuhira J, Tonosu J, Kasahara S, Tanaka S, et al. Association between somatic symptom burden and health-related quality of life in people with chronic low back pain. Minami M, editor. PLoS ONE. 2018 Feb 20;13(2):e0193208.

12. Angst F, Benz T, Lehmann S, Wagner S, Simmen BR, Sandòr PS, et al. Extended overview of the longitudinal pain-depression association: A comparison of six cohorts treated for specific chronic pain conditions. Journal of Affective Disorders. 2020 Aug;273:508–16.

13. Sima S, Lapkin S, Gan Z, Diwan AD. Association Between Non-spinal Comorbid Medical Conditions and Neuropathic Low Back Pain. – A Further Unravelling of Pain Complexities in the Context of Back Pain. Global Spine Journal. 2024 Aug 12;21925682241276441.

14. Tarabeih N, Kalinkovich A, Shalata A, Cherny SS, Livshits G. Deciphering the Causal Relationships Between Low Back Pain Complications, Metabolic Factors, and Comorbidities. JPR. 2022 Jan;Volume 15:215–27.

15. He C, Chen H, Guo L, Xu L, Liu Q, Zhang J, et al. Prevalence and factors associated with comorbid depressive symptoms among people with low back pain in China: A cross-sectional study. Front Psychiatry. 2022 Jul 25;13:922733.

16. Aroke EN, Jackson P, Overstreet DS, Penn TM, Rumble DD, Kehrer CV, et al. Race, Social Status, and Depressive Symptoms: A Moderated Mediation Analysis of Chronic Low Back Pain Interference and Severity. Clin J Pain. 2020 Sep;36(9):658–66.

17. Melo Cruz MC, Santeularia Verges MT, Rius Llorens C, Gich Saladich IJ, Català Puigbó E. Influencia de las comorbilidades en la intensidad del dolor en los pacientes con lumbalgia crónica. Medicina Clínica. 2022 Jul;159(2):73–7.

18. Lemes ÍR, Morelhão PK, Verhagen A, Gobbi C, Oliveira CB, Silva NS, et al. Does the Number of Comorbidities Predict Pain and Disability in Older Adults With Chronic Low Back Pain? A Longitudinal Study With 6- and 12-Month Follow-ups. Journal of Geriatric Physical Therapy. 2024 Jan;47(1):21–7.

19. Povieng B, Moses-Hampton M, Wu H. P58. Patterns of comorbidities and medical treatment in patients with chronic low back pain in Chicagoland. The Spine Journal. 2022 Sep;22(9):S153–4.

20. Gallagher RM, Mossey JM. (209) Impact of Co-Morbid Depression on Self-Reported Pain and Physical and Emotional Functioning in Low Back Pain Patients. Pain Medicine. 2008 Jul 7;2(3):242–242.

21. Hagen EM, Svensen E, Eriksen HR, Ihlebæk CM, Ursin H. Comorbid Subjective Health Complaints in Low Back Pain: Spine. 2006 Jun;31(13):1491–5.

22. Yang H, Hurwitz EL, Li J, de Luca K, Tavares P, Green B, et al. Bidirectional Comorbid Associations between Back Pain and Major Depression in US Adults. Int J Environ Res Public Health. 2023 Feb 27;20(5):4217.

23. De Luca KE, Parkinson L, Haldeman S, Byles JE, Blyth F. The Relationship Between Spinal Pain and Comorbidity: A Cross-sectional Analysis of 579 Community-Dwelling, Older Australian Women. Journal of Manipulative and Physiological Therapeutics. 2017 Sep;40(7):459–66.

24. Goode A, Cook C, Brown C, Isaacs R, Roman M, Richardson W. Differences in Comorbidities on Low Back Pain and Low Back Related Leg Pain. Pain Practice. 2011 Jan;11(1):42–7.

25. Fu Y, Chiarotto A, Enthoven W, Skou ST, Koes B. The influence of comorbidities on outcomes for older people with back pain: BACE-D cohort study. Annals of Physical and Rehabilitation Medicine. 2023 Oct;66(7):101754.

26. Parreira PCS, Maher CG, Ferreira ML, Machado GC, Blyth FM, Naganathan V, et al. A longitudinal study of the influence of comorbidities and lifestyle factors on low back pain in older men. Pain. 2017 Aug;158(8):1571–6.

27. Holmberg S, Thelin A, Stiernström EL, Svärdsudd K. Low back pain comorbidity among male farmers and rural referents: a population-based study. Ann Agric Environ Med. 2005;12(2):261–8.

28. Fernández-de-las-Peñas C, Alonso-Blanco C, Hernández-Barrera V, Palacios-Ceña D, Jiménez-García R, Carrasco-Garrido P. Has the prevalence of neck pain and low back pain changed over the last 5 years? A population-based national study in Spain. The Spine Journal. 2013 Sep;13(9):1069–76.

29. Emorinken A, Erameh CO, Akpasubi BO, Dic-Ijiewere MO, Ugheoke AJ. Epidemiology of low back pain: frequency, risk factors, and patterns in South-South Nigeria. Reumatologia. 2023 Oct 31;61(5):360–7.

30. Heuch I, Heuch I, Hagen K, Sørgjerd EP, Åsvold BO, Zwart JA. Is chronic low back pain a risk factor for diabetes? The Nord-Trøndelag Health Study. BMJ Open Diab Res Care. 2018 Oct;6(1):e000569.

31. Robertson D, Kumbhare D, Nolet P, Srbely J, Newton G. Associations between low back pain and depression and somatization in a Canadian emerging adult population. J Can Chiropr Assoc. 2017 Aug;61(2):96–105.

32. Badley EM, Millstone DB, Perruccio AV. Back Pain and Co-occurring Conditions: Findings From a Nationally Representative Sample. Spine. 2018 Aug;43(16):E935–41.

33. Haas R, Gorelik A, Busija L, O’Connor D, Pearce C, Mazza D, et al. Prevalence and characteristics of musculoskeletal complaints in primary care: an analysis from the population level and analysis reporting (POLAR) database. BMC Prim Care. 2023 Feb 4;24(1):40.

34. Stubbs B, Koyanagi A, Thompson T, Veronese N, Carvalho AF, Solomi M, et al. The epidemiology of back pain and its relationship with depression, psychosis, anxiety, sleep disturbances, and stress sensitivity: Data from 43 low- and middle-income countries. General Hospital Psychiatry. 2016 Nov;43:63–70.

35. Demyttenaere K, Bruffaerts R, Lee S, Posada-Villa J, Kovess V, Angermeyer MC, et al. Mental disorders among persons with chronic back or neck pain: Results from the world mental health surveys. Pain. 2007 Jun;129(3):332–42.

36. Gerhardt A, Hartmann M, Schuller-Roma B, Blumenstiel K, Bieber C, Eich W, et al. The prevalence and type of Axis-I and Axis-II mental disorders in subjects with non-specific chronic back pain: results from a population-based study. Pain Med. 2011/08/04 ed. 2011 Aug;12(8):1231–40.

37. Xu Y, Wang Y, Chen J, He Y, Zeng Q, Huang Y, et al. The comorbidity of mental and physical disorders with self-reported chronic back or neck pain: Results from the China Mental Health Survey. Journal of Affective Disorders. 2020 Jan;260:334–41.

38. iBrain - International Institute for the Brain, New York, NY, Pedro VM, Oggero E, Electrical and Computer Engineering Department, University of Wyoming, Laramie, WY, Vestibular Technologies, LLC, Cheyenne, WY. COMORBIDITY CHARACTERISTICS OF ADULT PATIENTS WITH REPORTED NECK AND LOW BACK COMPLAINTS - AN OUTPATIENT CLINICAL POPULATION-BASED COHORT STUDY. Biomed Sci Instrum. 2021 Apr 1;57(2):159–67.

39. Hartvigsen J, Natvig B, Ferreira M. Is it all about a pain in the back? Best Practice & Research Clinical Rheumatology. 2013 Oct;27(5):613–23.

40. Manchikanti L, Hirsch JA. What can be done about the increasing prevalence of low back pain and associated comorbid factors? Pain Management. 2015 May;5(3):149–52.

41. Meyer T, Wulff K. Issues of comorbidity in clinical guidelines and systematic reviews from a rehabilitation perspective. Eur J Phys Rehabil Med [Internet]. 2019 May [cited 2023 Mar 20];55(3). Available from: https://www.minervamedica.it/index2.php?show=R33Y2019N03A0364

42. Beeckmans N, Vermeersch A, Lysens R, Van Wambeke P, Goossens N, Thys T, et al. The presence of respiratory disorders in individuals with low back pain: A systematic review. Manual Therapy. 2016 Dec;26:77–86.

43. Øverås CK, Nilsen TIL, Nicholl BI, Rughani G, Wood K, Søgaard K, et al. Multimorbidity and co-occurring musculoskeletal pain do not modify the effect of the selfBACK app on low back pain-related disability. BMC Med. 2022 Dec;20(1):53.

44. Hestbaek L, Leboeuf-Yde C, Manniche C. Is low back pain part of a general health pattern or is it a separate and distinctive entity? A critical literature review of comorbidity with low back pain. Journal of Manipulative and Physiological Therapeutics. 2003 May;26(4):243–52.
