# Supplementary material for: Mapping Comorbidities in Patients With Low Back Pain—A Systematic Review
Source: Physiother Res Int. 2025 Sep 19;30(4):e70109. doi: 10.1002/pri.70109 (PMC12449286; doi:10.1002/pri.70109)
Supplement: Supplementary file 4 — Supporting Information S4: Extended information of included studies. [file PRI-30-e70109-s003.docx]

| **Appendix 4:** Extended information of included studies | | | | | | | |
| --- | --- | --- | --- | --- | --- | --- | --- |
| Study | Duration of LBP | Marital Status | Education | Employment | BMI | Smoking | Comorbidity Prevalence (%) |
| Rafn et al. (2023) | Acute < 6 weeks | N/A | University (11.5%) | Employed (82.1%) | <25.0 (41.2%) | Smoker (16.2%) | Hypertension (19%) |
|  |  |  | Medium education (27.4%) | Unemployed (17.9%) | 25.0-30.0 (37.5%) | Former smoker (34.2%) | Osteoarthritis (14.6%) |
|  |  |  | Short education (42%) |  | >30.0 (21.4%) | Never smoked (49.6%) | Migraine (8.7%) |
|  |  |  | None (15.5%) |  |  |  | Asthma (8.6%) |
|  |  |  | Other (3.7%) |  |  |  | Psoriasis (5%) |
|  |  |  |  |  |  |  | Metabolic diseases (4.7%) |
|  |  |  |  |  |  |  | Stroke (3.9%) |
|  |  |  |  |  |  |  | Cancer (3.8%) |
|  |  |  |  |  |  |  | Diabetes (3.5%) |
|  |  |  |  |  |  |  | COPD/Chronic bronchitis (2%) |
|  |  |  |  |  |  |  | Osteoporosis (1.6%) |
|  |  |  |  |  |  |  | Rheumatoid arthritis (1.6%) |
|  |  |  |  |  |  |  | Inflammatory bowel disease (1.3%) |
|  |  |  |  |  |  |  | Fibromyalgia (0.4%) |
|  |  |  |  |  |  |  | Neurological disease (0.8%) |
| Gore et al. (2012) | Chronic > 12 weeks | N/A | N/A | N/A | N/A | N/A | Chronic back and neck pain (besides LBP) (43%) |
|  |  |  |  |  |  |  | Other musculoskeletal pain conditions (41%) |
|  |  |  |  |  |  |  | Rheumatism (excluding the back) (40%) |
|  |  |  |  |  |  |  | Arthritis and other joint diseases (34%) |
|  |  |  |  |  |  |  | Back and neck pain with neuropathic involvement (excluding lower back) (34%) |
|  |  |  |  |  |  |  | Neuropathic LBP (30.6%) |
|  |  |  |  |  |  |  | Osteoarthritis (14%) |
|  |  |  |  |  |  |  | Depression (13%) |
|  |  |  |  |  |  |  | Insomnia/sleep disorders (10%) |
|  |  |  |  |  |  |  | Anxiety (8%) |
|  |  |  |  |  |  |  | Other polyneuropathies (3.9%) |
|  |  |  |  |  |  |  | Carpal tunnel syndrome (3.1%) |
|  |  |  |  |  |  |  | Rheumatoid arthritis (1.7%) |
|  |  |  |  |  |  |  | Pain syndromes (1.4%) |
|  |  |  |  |  |  |  | Diabetic neuropathy (0.4%) |
|  |  |  |  |  |  |  | Connective tissue diseases (0.3%) |
|  |  |  |  |  |  |  | Atypical facial pain (0.2%) |
|  |  |  |  |  |  |  | Autonomic neuropathies (0.2%) |
|  |  |  |  |  |  |  | Trigeminal neuralgia (0.1%) |
|  |  |  |  |  |  |  | Postherpetic neuralgia (0.1%) |
|  |  |  |  |  |  |  | Phantom pain (0%) |
| Schneider et al. (2007) | Any duration | Married (66%) | >13 years (28%) | Full-time (33.3%) | N/A | N/A | Osteoarthritis (33%) |
|  |  | Divorced (9.2%) | 13 years (27.6%) | Part-time (35.6%) |  |  | Gastritis (30%) |
|  |  | Single (18.2%) | 10 years (35.8%) | Unemployed/retired (38.1%) |  |  | Hypertension (26%) |
|  |  | Widowed (6.6%) | 9 years (39.7%) |  |  |  | Varicose veins (25%) |
|  |  |  | No education (34.4%) |  |  |  | Migraine (23%) |
|  |  |  |  |  |  |  | Contact dermatitis (23%) |
|  |  |  |  |  |  |  | Inflammatory joint or spine disease (e.g., rheumatoid arthritis) (22%) |
|  |  |  |  |  |  |  | Thyroid disorder (18%) |
|  |  |  |  |  |  |  | Allergic rhinitis or conjunctivitis (18%) |
|  |  |  |  |  |  |  | Colitis or gallstones (13%) |
|  |  |  |  |  |  |  | Chronic bronchitis (12%) |
|  |  |  |  |  |  |  | Pyelonephritis (11%) |
|  |  |  |  |  |  |  | Urticaria (11%) |
|  |  |  |  |  |  |  | Perfusion disturbances in the heart (10%) |
|  |  |  |  |  |  |  | Perfusion disturbances in the legs (10%) |
|  |  |  |  |  |  |  | Peptic ulcer (10%) |
|  |  |  |  |  |  |  | Asthma (9%) |
|  |  |  |  |  |  |  | Kidney stones (9%) |
|  |  |  |  |  |  |  | Osteoporosis (9%) |
|  |  |  |  |  |  |  | Heart failure (7%) |
|  |  |  |  |  |  |  | Hepatitis (7%) |
|  |  |  |  |  |  |  | Food allergy (7%) |
|  |  |  |  |  |  |  | Atopic eczema (6%) |
|  |  |  |  |  |  |  | DVT (5%) |
|  |  |  |  |  |  |  | Diabetes mellitus (non-insulin dependent) (5%) |
|  |  |  |  |  |  |  | Cancer (5%) |
|  |  |  |  |  |  |  | Myocardial infarction (3%) |
|  |  |  |  |  |  |  | Cerebrovascular perfusion disorder (3%) |
|  |  |  |  |  |  |  | Stroke (2%) |
|  |  |  |  |  |  |  | Diabetes mellitus (insulin dependent) (2%) |
|  |  |  |  |  |  |  | Meningitis (2%) |
| Ramanathan et al. (2018) | Any duration | N/A | <10 years (29.9%) | Employed (36.5%) | N/A | N/A | Hypertension (28.7%) |
|  |  |  | High school (15.8%) | Unemployed (1.7%) |  |  | Osteoarthritis (25.6%) |
|  |  |  | Professional education (37.7%) | Retired (51.5%) |  |  | Hyperlipidemia (15.9%) |
|  |  |  | University (16.6%) | Student/other (10.3%) |  |  | Dyspepsia (13.4%) |
|  |  |  |  |  |  |  | Depression (9.8%) |
|  |  |  |  |  |  |  | Overweight (9.1%) |
|  |  |  |  |  |  |  | Osteoporosis (7.9%) |
|  |  |  |  |  |  |  | Atherosclerosis (7.3%) |
|  |  |  |  |  |  |  | Diabetes (5.5%) |
|  |  |  |  |  |  |  | Asthma (4.9%) |
| Leopoldino et al. (2020) | Acute < 6 weeks | Married (44.2%) | High school or higher (37.3%) | N/A | Mean: 28.9 (5.2) | N/A | Hypertension (70.9%) |
|  |  | Unmarried (55.8%) | Lower than high school (62.7%) |  |  |  | Osteoarthritis (46.9%) |
|  |  |  |  |  |  |  | Gastric disease/ulcer (33.6%) |
|  |  |  |  |  |  |  | Depression (32.7%) |
|  |  |  |  |  |  |  | Diabetes (23.8%) |
| Ritzwoller et al. (2006) | Any duration | N/A | N/A | N/A | N/A | N/A | Hypertension (19.4%) |
|  |  |  |  |  |  |  | Inflammation (15.6%) |
|  |  |  |  |  |  |  | Heart disease/hypertension (14%) |
|  |  |  |  |  |  |  | Depression (13.4%) |
|  |  |  |  |  |  |  | Anxiety (12.2%) |
|  |  |  |  |  |  |  | Gastrointestinal disease (12.1%) |
|  |  |  |  |  |  |  | Asthma/COPD (12%) |
|  |  |  |  |  |  |  | Thyroid disorder (7.9%) |
|  |  |  |  |  |  |  | Vascular disease (6.8%) |
|  |  |  |  |  |  |  | Hyperlipidemia (6.2%) |
|  |  |  |  |  |  |  | Diabetes (5.2%) |
|  |  |  |  |  |  |  | Rheumatoid arthritis (4.4%) |
|  |  |  |  |  |  |  | Ischemic cardiovascular disease (3%) |
|  |  |  |  |  |  |  | Psychosis (2.4%) |
| Bartholomeeusen et al. (2012) | Any duration | N/A | N/A | N/A | N/A | N/A | Neck pain (5.7%) |
|  |  |  |  |  |  |  | Shoulder syndrome (0.7%) |
|  |  |  |  |  |  |  | Other musculoskeletal disorders (0.4%) |
|  |  |  |  |  |  |  | Hip osteoarthritis (0.2%) |
|  |  |  |  |  |  |  | Depression (0.2%) |
| Korff et al. (2005) | Chronic > 12 weeks | N/A | ≥16 years (26.2%) | Employed (26.4%) | N/A | N/A | Osteoarthritis (51%) |
|  |  |  | 13-15 years (27.3%) | Student (13.4%) |  |  | Hypertension (26.6%) |
|  |  |  | 12 years (30.5%) | Homemaker (29.1%) |  |  | Other chronic pain (18.9%) |
|  |  |  | 0-11 years (34.5%) | Retired (38.3%) |  |  | Asthma (16.9%) |
|  |  |  |  | Unemployed/disabled (41.5%) |  |  | Other headaches (14.6%) |
|  |  |  |  |  |  |  | Migraine (12.5%) |
|  |  |  |  |  |  |  | Diabetes/high blood sugar (8.2%) |
|  |  |  |  |  |  |  | Vision problems (7.1%) |
|  |  |  |  |  |  |  | Heart disease (6.9%) |
|  |  |  |  |  |  |  | Peptic ulcer (6.6%) |
|  |  |  |  |  |  |  | Hearing problems (6%) |
|  |  |  |  |  |  |  | Stroke (4.3%) |
|  |  |  |  |  |  |  | Other lung diseases (4.1%) |
|  |  |  |  |  |  |  | Heart attack (4%) |
|  |  |  |  |  |  |  | Epilepsy (3%) |
|  |  |  |  |  |  |  | Irritable bowel syndrome (1.9%) |
|  |  |  |  |  |  |  | Cancer (0.8%) |
|  |  |  |  |  |  |  | HIV (0.3%) |
| Marunica Karšaj et al. (2023) | Any duration | Married (67.2%) | College/University (21.3%) | Working/student (31.3%) | <25.0 (30.5%) | N/A | Chronic neck pain (65.7%) |
|  |  | Never married (7.8%) | High school (53.4%) | Unemployed (6.3%) | 25.0-30.0 (41%) |  | Hypertension (42.9%) |
|  |  | Widowed/Divorced/Single (25%) | Elementary school (25.4%) | Retired/inactive (62.3%) | >30.0 (28.6%) |  | Allergies (23.9%) |
|  |  |  |  |  |  |  | Osteoarthritis (19.8%) |
|  |  |  |  |  |  |  | Urinary incontinence (19.0%) |
|  |  |  |  |  |  |  | Diabetes mellitus (14.2%) |
|  |  |  |  |  |  |  | Coronary heart disease (13.1%) |
|  |  |  |  |  |  |  | Kidney problems (12.7%) |
|  |  |  |  |  |  |  | Depression (11.9%) |
|  |  |  |  |  |  |  | Chronic bronchitis (10.5%) |
|  |  |  |  |  |  |  | Stroke (8.2%) |
|  |  |  |  |  |  |  | Heart attack (6.7%) |
|  |  |  |  |  |  |  | Asthma (3.4%) |
|  |  |  |  |  |  |  | Liver cirrhosis (1.9%) |
| LBP = Low back pain; N/A = not available | | | | | | | |
