# Supplementary material for: Mapping Comorbidities in Patients With Low Back Pain—A Systematic Review
Source: Physiother Res Int. 2025 Sep 19;30(4):e70109. doi: 10.1002/pri.70109 (PMC12449286; doi:10.1002/pri.70109)
Supplement: Supplementary file 5 — Supporting Information S5: Level of certainty in comorbidity prevalence estimates in included studies. [file PRI-30-e70109-s001.docx]

**Appendix 5** Level of certainty in comorbidity prevalence estimates in included studies

| **Study** | **Certainty of Prevalence Estimates** | **Justification** |
| --- | --- | --- |
| Rafn et al. (2023) | Low Certainty | The study relies entirely on self-reported data, which introduces recall bias; however, the use of validated questionnaires increases reliability. Additionally, the study is limited to private settings (chiropractic care), which reduces its generalizability to broader populations. |
| Gore et al. (2012) | Low Certainty | The study includes only insured individuals in the U.S., which limits representativeness and introduces selection bias, as individuals without insurance may have different health profiles. |
| Schneider et al. (2007) | Low Certainty | Based on a nationally representative health survey however with moderate response rate 61,4%), which introduces some risk of selection bias. Self-reported data validated by physician diagnoses reduces recall bias. |
| Ramanathan et al. (2018) | Very Low Certainty | The study has a small sample size (n=164) and very high attrition, making it uncertain whether the comorbidity profile is broad and representative. |
| Leopoldino et al. (2020) | Very Low Certainty | The study uses a convenience sample, leading to selection bias since participants were volunteers rather than randomly selected. Self-reported data increases the risk of recall bias, and no comparison with objective measures was made. |
| Ritzwoller et al. (2006) | Low Certainty | The study uses a large database with validated diagnosis codes, reducing reporting bias. However, it only includes insured patients, meaning those without insurance (potentially with different comorbidity burdens) are excluded, limiting generalizability. |
| Bartholomeeusen et al. (2012) | Moderate Certainty | The study is based on medical records and diagnosis codes, minimizing recall bias. However, it is limited to a single region in Belgium, which may affect generalizability. |
| Von Korff et al. (2005) | Very Low Certainty | The study relies entirely on self-reported questionnaires, leading to high recall bias. Selection bias is likely, as the study disproportionately includes individuals with psychological comorbidities, making the findings less generalizable to the overall LBP population. The study is based in the U.S., where healthcare access disparities may further distort prevalence estimates. |
| Marunica Karšaj et al. (2023) | Moderate Certainty | The study relies on self-reported data, which introduces recall bias, but it benefits from a strong methodological design. It uses a large, stratified random sample with a high response rate (83%), which enhances the certainty of comorbidity estimates. |
